# Doctors’ duty to provide abortion information

**DOI:** 10.1093/jlb/lsad024

**Published:** 2023-09-01

**Authors:** Michelle Oberman, Lisa Soleymani Lehmann

**Keywords:** abortion, reproductive justice, bioethics

## Abstract

With abortion remaining legal in over half of the country and a proliferation of websites offering information on how to access abortion medications, for those who know where to look, there are sound options for safely ending an unwanted early-stage pregnancy. But not all patients have equal access to reliable information. This Article addresses the urgent downstream harms caused by the lack of access to abortion information, and argues that in view of these consequences, regardless of abortion’s legal status, clinicians have a duty to provide their patients with abortion information. We begin by documenting clinicians’ hesitation to share abortion information, drawing on our interviews with 25 doctors practicing medicine in a state where abortion is criminalized. Next, we explain why clinicians are duty-bound to provide all-options counseling. We then consider whether such duties shift where abortion is criminalized. After identifying the limited legal risks associated with supplying abortion information, and showing how, by requiring all-options counseling, professional societies might reduce risks to patients and clinicians, we conclude that, regardless of the legal status of abortion, clinicians have a professional responsibility to share basic abortion information – including treatment options and how to access those options.

## I. INTRODUCTION

With abortion remaining legal in over half of the country and a proliferation of websites offering practical support and information on how to access abortion medications regardless of a person’s US state of residence, ending an unwanted first-trimester pregnancy can be readily accomplished in spite of abortion bans. But not all patients have equal access to reliable information. In 2022, the World Health Organization (WHO), which has been issuing abortion-related guidelines since 2003, listed the scarcity of accurate information first among the abortion-related problems that jeopardize sexual and reproductive well-being and health.^1^ Long before the Dobbs decision permitted states to criminalize abortion, the most marginalized US patients—in particular, low-income, first-generation immigrants and Spanish speakers—lacked accurate knowledge about how to access abortion.^2^ In today’s complicated legal climate, similarly vulnerable patients might need their doctors’ help to identify trustworthy abortion information.^3^ Yet, our research suggests healthcare providers are often hesitant to provide it.

We are not talking about providers who opt out of abortion-related care as conscientious objectors, refusing to share abortion information because they believe it implicates them in what they view as morally objectionable behavior. We leave for another day the myriad ethical questions raised by conscientious objector status in an era of abortion bans.^4^ Here, we are concerned solely with clinicians who hesitate to provide basic abortion information because they fear the professional risks and legal consequences of doing so. We argue that healthcare providers have an affirmative obligation to inform patients about their options for abortion care. Remaining silent and not informing patients about their abortion options violate ethical obligations and professional norms.

We begin with an overview of the findings from interviews with 25 doctors practicing medicine in a state where the provision of abortion is criminalized. Using these interviews as a springboard for our analysis, we then examine clinicians’ ethical and professional obligations in the context of providing abortion information, showing how the provision of abortion information is central to sound ethical and clinical practice. Following this analysis, we consider the question of whether these duties shift where abortion is criminalized. After identifying the limited legal risks associated with supplying abortion information, and showing how, by requiring all-options counseling, professional societies might reduce risks to patients and clinicians, we conclude that, regardless of the legal status of abortion, clinicians have a professional responsibility to share basic abortion information – including treatment options and how to access those options. 

## II. RESEARCH FINDINGS

In July 2022, just days after the Supreme Court’s *Dobbs*^5^ decision permitted states to criminalize abortion, we launched a research project to study the impact of a new abortion ban on one state’s clinicians. In order to protect our participants, in addition to the Food and Drug Administration's (FDA) Institutional Review Board’s approval,^6^ we obtained a National Institute of Health Certificate of Confidentiality^7^ and worked with hospital counsel to establish a protocol that fully anonymized their identities and their location. Therefore, we refer to the site of our research project only as a mid-sized US city (approximately 3 million people), in a state where abortion is banned outside of a narrow exception for life-threatening medical emergencies. We chose the location for our research based on several factors: a state government with an activist anti-abortion agenda; the presence of nationally ranked hospitals; the availability of legal abortion in some neighboring states; and a diverse urban setting with entrenched poverty and racism, reflected both in terms of a history of racial conflict and in factors like high rates of Black maternal mortality and a resistance to Medicaid expansion and other social welfare policies. What interested us in these latter factors is the extent to which they reflect a relative indifference to the forces contributing to higher abortion rates among poor, disproportionately Black and brown Americans.^8^

We conducted a series of semi-structured hour-long interviews with 25 doctors in a range of practice areas (addiction medicine, adolescent medicine, emergency medicine, reproductive endocrinology, and maternal–fetal medicine), and a diversity of practice settings (academic medical centers, private practice, and religiously affiliated hospitals).^9^ Although all of the participants routinely encountered patients who needed care related to pregnancy termination, only two of the participants worked specifically as abortion providers. Prior to the abortion ban, the remainder had made referrals to local providers when patients sought routine abortion care.

It was hard to find doctors willing to be interviewed. Our initial outreach strategy entailed asking trusted colleagues from around the country in law, health care, and medical ethics, as well as contacts from our personal networks, to help connect us with local providers who might be willing to be interviewed. Prior to our research trip, we contacted 30 clinicians, of whom nine either declined to be interviewed or did not respond to our request for an interview. Once we arrived, we employed a snowball methodology to expand our pool of participants, adding four more interviews to our pool.^10^

There is one notable category missing from our study: despite intense effort, we were unable to find a single self-identified ‘pro-life’ clinician willing to meet with us. Although they varied in the strength of their personal position with respect to whether they believed abortion was moral, each of the doctors we interviewed opposed the abortion ban and thought it should be legal to end an unwanted pregnancy. As our participants were not conscientious objectors to abortion-related care, they provide a window into the ways in which abortion bans can impact the clinical practice of physicians who think their patients should be able to choose abortion.

All but two of the interviews were in-person, and each followed a scripted set of open-ended questions designed to illuminate the ways in which the abortion ban had altered pregnancy care generally and abortion-related care in particular.

We arrived to find clinicians shell-shocked. It quickly emerged that our participants were concerned (some intensely so) about the risks of running afoul of the ban. They worried about prosecution,^11^ civil liability, losing a job, their medical license, and a livelihood.^12^ Participants also described the law as having impacted patient care at a quieter level than the cases that tend to make national headlines, such as those involving miscarriage management or fatal fetal anomalies.^13^ Criminalizing the provision of abortion had complicated the way doctors were responding to patients who wanted or needed an abortion.

Even prior to *Dobbs*, informed consent in the abortion context was already ethically contested territory for these clinicians. Prior to the ban, the state law required doctors to give all patients seeking abortion a booklet informing them of ‘facts’ that included discredited theories surrounding the risks of abortion, as well as those simply not amenable to proof, such as assertions about when life begins.^14^ Several of our clinicians described a long-standing practice of acting as a trusted healthcare intermediary when delivering the brochure, assuring their patients that, although the state required them to deliver the pamphlet, they disagreed with some of the assertions it contained, and were open to answering, without judgement, any questions they had about abortion.^15^

In the eyes of the clinicians we interviewed, criminalizing abortion ratcheted up the risks associated with having abortion conversations with patients, driving a reticence that was at odds with their understanding that, to have integrity as a healthcare provider, they should not be withholding medical information and needed to continue having fact-based conversations about abortion. As one of our participants explained:

I think the issue that really keeps me awake at night … is having regulations about what I can counsel a patient about. [A]s it stands, it gives me some comfort [if] I can tell a patient, at least like, here are the places you can go to get this done. But what I worry about is the inability to even offer that to a patient and to say, like, ‘Your baby has a lethal anomaly, sorry.’ And that’d be the end of the conversation. That really, that keeps me up at night.^16^

Others were under the impression that they were being gagged and could not provide any abortion information at all. One participant said, “Based on the way the law is written … our counsel at our hospital was concerned that we can’t even … refer a patient. So, I can’t even tell her, you can call the [clinic in neighboring state] at whatever their number is there.”^17^

Most of our providers said they were telling or planning to tell patients that abortion was legal in nearby states. But few described going beyond that, to address the questions their patients are likely to have about how they can still access an abortion. As one participant said:

[The question is] how we can still provide full options. Like what’s the most efficient way to connect someone who needs a termination? And then what are charity services that could help provide funding for people who need funding. And then transportation… because of course who will get left behind again, you know, are our patients from rural areas … [those] that are far from any service and then people who are poor.^18^

The doctors we met already knew which of their patients would struggle as a result of their failure to have a more detailed conversation. As one doctor said:

We see a pretty diverse patient population across the whole socio-economic and educational background. And I would say the majority of my patients are going to have difficulty navigating that. Because they can’t, you know, pull out your smartphone and get on the internet. You know, scroll through all the filters and algorithms. But not everybody has the internet at home. Not everybody has reliable transportation to get somewhere. Not everybody has a safe place they can make that phone call from.^19^

And yet, their fear of the law left many feeling that they had little choice but to restrict the information they provided. Indeed, the same doctor said he would counsel patients using the following language: “There are other states with different options, but that’s something you’d have to explore because I’m not allowed to refer you there, based on the law.”^20^

Because we conducted these interviews so soon after abortion became illegal, it is possible that our interviewees were over-correcting in the face of changed circumstances and that with time, they will return to a practice of providing comprehensive abortion information to their patients.^21^ But we are dubious about that prospect, in large part because their concerns were driven by the fact that they were uncertain about the legality of sharing abortion information. Without clear resolution and guidance from the profession, it is highly likely that they will continue pulling back from potential personal risk, a classic illustration of the so-called ‘chilling effect’ of the law.^22^ This concern is borne out by findings from a June 2023 Kaiser Family Foundation survey of obstetricians and gynecologists practicing in states with abortion bans. It found that 78 per cent were unwilling to refer patients for out-of-state, legal abortions, and 30 per cent failed to even offer their patients abortion information such as online resources.^23^

It is easy to understand the chilling effect of laws criminalizing the provision of abortion. The doctors we met were at various stages of their careers, some shouldering massive student loan debt, others raising families. They were hoping to avoid becoming the first doctor prosecuted, or the first to lose their license.^24^ But as we explain in the next section, the doctor–patient relationship cannot be guided by a fear of liability. Instead, clinicians are bound by fundamental ethical and professional obligations to provide their patients with basic abortion information.

## III. ABORTION AND THE ETHICAL AND PROFESSIONAL DUTY TO PROVIDE HEALTH INFORMATION

Regardless of its legal status, abortion care is a core component of comprehensive reproductive health care. After explaining why this is so, both generally, and in particular regarding abortion information, this section examines the ethical and professional obligations that apply to the provision of abortion care.

### III.A. Abortion Information as Comprehensive Reproductive Health Care

Abortion care is an essential part of comprehensive reproductive health care. In the words of the WHO’s Abortion Care Guideline Development Group, abortion care “includes information provision, abortion management, and post-abortion care, is an integral component of sexual and reproductive health and is a safe, simple health-care intervention that saves women’s lives and safeguards their dignity and bodily autonomy.”^25^ The leading US medical organizations in the reproductive field agree, recognizing induced abortion—the intentional medical or surgical termination of a pregnancy—as ‘an essential component of women’s health care’.^26^

The centrality of abortion to comprehensive reproductive health care also stems from the significant, negative health consequences that result when people lack access to safe abortion. In explaining why induced abortion is ‘an essential component of women’s health care’, the American College of Obstetricians and Gynecologists (ACOG) notes:

“Where abortion is illegal or highly restricted, women resort to unsafe means to end unwanted pregnancies, including self-inflicted abdominal and bodily trauma, ingestion of dangerous chemicals, self-medication with a variety of drugs, and reliance on unqualified abortion providers.”^27^

Historically, where legally restricted, abortion was associated with high rates of morbidity and mortality.^28^ In the past two decades, the advent of medication abortion has made it possible for people to safely self-manage an abortion prior to a gestational age of 12 weeks.^29^ Abortion-related maternal deaths have plummeted worldwide as access to information about medication abortion has spread.^30^ Rather than struggle to find someone to perform a surgical abortion, a person can read about and purchase abortion pills online and then end their pregnancy in the privacy of their home.

The reality is that access to safe abortion today turns in large part on access to reliable abortion information. Without access to accurate abortion information, patients face the elevated risks of negative health outcomes that have historically been associated with illegal abortion.^31^ According to the WHO, unsafe abortions cause 39,000 deaths a year, along with millions of hospitalizations.^32^ Other patients may incorrectly believe they have no alternative but to carry to term. They will endure the risks of pregnancy—far greater than those of abortion^33^—along with the mental health consequences of forced pregnancy, forced child-bearing, and, for the overwhelming majority, the life-altering consequences of child-rearing.^34^

In view of these risks, there are several reasons why doctors have an obligation to share abortion information. First, human rights law guarantees a right to information, which has been held to extend to patients seeking access to abortion information. In a 1992 decision growing out of the Irish government’s attempt to restrict abortion information, the European Court of Human Rights ruled that the government could not prohibit counselors from informing people in Ireland about lawful abortion services available in England.^35^ They found such censorship violated the individual’s freedom to receive and impart information.^36^ Second, access to abortion information may be understood as a vital harm-reduction strategy, both at the individual and at the population level. In her 2011 article, *Access to Information on Safe Abortion*, Professor Joanna Erdman develops both perspectives, making a case for promoting access to abortion information both as a matter of human rights and sound public health policy.^37^

In our view, though, the most compelling arguments for providing abortion information arise out of medical ethics and professional norms. As we explain below, clinicians are duty-bound to promote their patients’ well-being, to empower patients to make medical decisions consistent with their own values, and to refrain from doing anything that will harm their patients. All of these obligations lead to the inescapable conclusion that doctors not only have permission but also have an ethical and professional duty to share abortion information with patients.

### III.B. Bioethical Underpinnings of the Duty to Provide Abortion Information

Clinicians who fail to share basic abortion information where relevant to their patients’ treatment options contravene the central ethical obligations of the medical profession: autonomy, beneficence, non-maleficence, and justice.^38^ The obligation to respect and promote patient autonomy—the lynchpin of modern medical ethics—is a core justification for clinicians’ broad ethical duty to provide health information.^39^ Generally speaking, inadequate or inaccurate health information undermines patients’ autonomy by imperiling their ability to make an informed decision, consistent with their values. Therefore, clinicians have an ethical obligation to communicate health information in a manner that empowers their patients to make informed decisions about their treatment.

From this perspective, access to abortion information emerges as central to the clinician’s ethical obligation to promote patient autonomy. Such information is vital to a patient’s ability to chart their own life course. Indeed, it is hard to imagine a type of health information more closely tied to patient autonomy. By failing to provide abortion information, a doctor effectively deprives their patient of the range of options available to those who enjoy enough privilege to be able to access and understand information about how to end an unwanted pregnancy.

Equally, the duty to share abortion information is grounded in the twin ethical injunctions of beneficence and non-maleficence, giving rise to the obligation to prioritize the patient's best interests, promote their well-being, and act for their benefit.^40^ Broadly speaking, the beneficence-based case for a duty to provide health information arises because patients who lack adequate health information experience heightened rates of negative health outcomes.^41^ Consequently, doctors are duty-bound to communicate in a manner that ensures their patients understand the information they need in order to safeguard their own health and well-being.^42^ Because the paramount aim of the beneficent provider is to protect and promote their patient’s well-being, clinicians are duty bound to provide accurate and comprehensive abortion information, and must do so in a manner that ensures their patients understand their abortion-related options, regardless of abortion’s legal status.

Furthermore, doctors’ silence places their most vulnerable patients—particularly poor people of color—at increased risk of negative health outcomes. Withholding abortion information is therefore at odds with physicians’ ethical obligation of non-maleficence--the duty to not harm patients. Patients lacking accurate information are more likely to delay, attempting abortion later in pregnancy, and using riskier, less effective methods. Consequently, they are at a heightened risk for medical complications. And when things go wrong, they are at heightened risk of prosecution, which is known to disproportionately target poor Black and brown women.^43^

This disparate impact on marginalized populations triggers the ethical injunction to promote justice. Broadly speaking, this principle is concerned with assuring a rational, fair, and equitable allocation of health resources for the greater good of society.^44^ Health information is relevant here because stratified access to and understanding of health information is costly not just for the individual but also for the population as a whole. Indeed, the downstream consequences of inadequate comprehension of health information include a financial drain on both individuals and society and an intensification of social inequity.^45^ Health literacy is so vital to patients’ well-being that it is the central focus of the US government’s Healthy People 2030 goals, which aim to “eliminate health disparities, achieve health equity, and attain health literacy to improve the health and well-being of all.”^46^

Low abortion literacy is costly on both an individual and a societal level. Patients who are vulnerable for reasons of poverty, race, geography, and age are disproportionately likely to struggle accessing and understanding all health information, including abortion information.^47^ Crucially, this is the same segment of the population that is most likely to experience an unwanted pregnancy^48^ and most likely to seek abortions.^49^

The results of being deprived of the information needed to obtain a wanted abortion include the intensification of poverty and a worsening of physical health outcomes for the pregnant person, their existing children, and the children born as a consequence of being denied an abortion.^50^ These harms also carry an intergenerational impact because one consequence of the failure to provide abortion information is to increase the number of children born into poverty. The result is what the American Academy of Pediatrics (AAP) calls the ‘medicalization of poverty’:

Children who experience poverty, particularly during early life or for an extended period, are at risk of a host of adverse health and developmental outcomes through their life course. Poverty has a profound effect on specific circumstances, such as birth weight, infant mortality, language development, chronic illness, environmental exposure, nutrition, and injury. Child poverty also influences genomic function and brain development. […] Children living in poverty are at increased risk of difficulties with self-regulation and executive function, such as inattention, impulsivity, defiance, and poor peer relationships. […] Child poverty is associated with lifelong hardship. Poor developmental and psychosocial outcomes are accompanied by a significant financial burden, not just for the children and families who experience them but also for the rest of society.^51^

Rather than being an abstraction, doctors’ duty to promote justice applies with particular force in the context of treating patients who are likely to struggle to identify accurate abortion information (eg those with low health literacy, low socioeconomic status, and lack of internet access). Indeed, because lack of access to accurate abortion information carries such powerful downstream consequences, it might be seen as the quintessential illustration of the importance of providing accurate health information as both a public health and an ethical intervention.

Any one of these core ethical principles would suffice to establish the duty to provide patients with abortion information. That each of these values is implicated simply underscores the fact that clinicians have an ethical obligation to inform patients about their treatment options.

### III.C. Professional Norms Governing the Duty to Provide Abortion Information

In addition to being unethical, there are strong professional norms that speak to the duty to provide abortion information. The leading professional medical societies support the position that abortion, and the provision of abortion information, is an essential component of comprehensive reproductive health care. In 2018, the American Academy of Family Practitioners (AAFP), the American Academy of Pediatrics (AAP), ACOG, and the American College of Physicians (ACP) issued Joint Principles on Protecting Physician-Patient Relationship, declaring that they:

Reject government restrictions on the information our patients can receive from their doctors. Patients expect medically accurate, comprehensive information from their physicians; this dialogue is critical to ensuring the integrity of the patient-physician relationship. No governmental body should interfere in our members’ obligation to provide evidence-based information to their patients. When our government restricts the information that can be given to women, or forces physicians to provide women with non-medically inaccurate information, we can expect increased rates of unplanned pregnancy, pregnancy complications, and undiagnosed medical conditions.^52^

Likewise, ACOG issue a statement declaring:

ACOG supports every person’s right to decide whether to have children, the number and spacing of children, and to have the information, education, and access to health services to make these decisions. Individuals seeking abortion must be afforded privacy, dignity, respect, and support, and should be able to make their medical decisions without undue interference by outside parties.^53^

In a 2022 Joint Statement, the country’s leading cancer organizations [the American Society of Clinical Oncology (ASCO) and Leukemia and Lymphoma Society (LLS)] underscored the professional norm requiring abortion information:

Every patient with cancer should receive evidence-based information about all treatment options, including known side effects of those options. Every patient should be able to maximize their chance for survival by receiving recommended care promptly.^54^

The American College of Emergency Physicians (ACEP) echoed the position of these societies, passing this Council Resolution in 2023:

RESOLVED, That ACEP supports the position that the early termination of pregnancy (publicly referred to as ‘abortion’) is a medical procedure, and as such, involves shared decision making between patients and their physician regarding: 1) discussion of reproductive health care; 2) performance of indicated clinical assessments; 3) evaluation of the viability of pregnancy and safety of the pregnant person; 4) availability of appropriate resources to perform indicated procedure(s); and 5) is to be made only by health care professionals with their patients.^55^

In addition to the professional norm that speaks directly to a duty to provide abortion information, when doctors fail to provide abortion information, thereby diminishing the quality of care they provide due to their worry about potential legal consequences, they violate a strong professional norm against permitting extraneous concerns to undermine sound medical practice.^56^ This norm is central to ensuring the integrity of the medical profession. A clinician who pulls back from sound medical practice in order to reduce legal risks to themselves effectively allocates to the state the responsibility for their individual medical decisions. Once the state is invited into the doctor–patient relationship, the lines of loyalty (ie to their patient or to the state) become blurry.

The result is a role confusion that, in the context of reproductive health care, has given rise to a concerning collusion between health care providers and law enforcement. The most vivid example of such divided loyalties is seen in the pattern of prosecutions of poor, largely minority women for alleged crimes arising out of miscarriages, stillbirths, or perceived risks taken while pregnant. Building on an earlier study from 2013, Pregnancy Justice, an organization providing legal defense to those charged with such crimes, has documented more than 1600 US women who have been prosecuted since 1973.^57^ Of these, 1200 occurred in the past 15 years alone.^58^ The prosecutions overwhelmingly target poor people, and poor, Black pregnant women in particular. Of 413 cases arising from 1973 to 2005, 71 per cent involved low-income women; 59 per cent were women of color, with 52 per cent identifying as Black.^59^

The typical case arises when clinicians notify police, in violation of legal and ethical norms safeguarding patient confidentiality.^60^ In Fall 2021, just weeks after Texas’ S.B. 8 was permitted to go into effect, effectively ending access to legal abortion in the state, we saw evidence of this pattern when a doctor called the police after his hemorrhaging patient told him she had taken abortion pills.^61^ The charges later were dropped because Texas law prohibits bringing abortion or homicide charges against those who end their own pregnancies,^62^ but it is hard to estimate the lingering damage, both to Ms. Herrera and also to the public’s perception of medical confidentiality.

For decades, professional medical societies have decried the practice of breaching confidentiality by reporting patients to police, noting that it harms patients, both by discouraging them from seeking medical care.^63^ It may seem like there is a meaningful difference between collaborating with police and simply remaining silent when a patient seeks abortion information, but the clinician who remains silent rather than supplying the information needed to safeguard a patient’s well-being effectively becomes an arm of the state, enforcing the broadest interpretation of the criminal law at the expense of their patient’s well-being.

For all of these reasons, ethical and professional norms plainly require clinicians to share abortion information. The only question is what clinicians should do when they fear that adhering to their professional ethics may violate the law. We turn to this question in the next section.

## IV. CONFRONTING THE RISKS OF PROVIDING ABORTION INFORMATION

Having established that the duty to provide abortion information is rooted in both ethical and professional norms, in this section we examine the legal risks faced by clinicians who do so when they reside in a state with abortion restrictions. We begin by identifying and evaluating the potential criminal and civil consequences of sharing abortion information, then turn to the question of how the profession might help minimize risks to individual clinicians.^64^

### IV.A. The Legal Risks of Providing Abortion Information^65^

The doctors we met spoke about a range of potential concerns surrounding the provision of abortion information, including ‘getting sued, getting arrested, having a record, losing [one’s] license’.^66^ At the time of our interviews—the first month after the law criminalizing the provision of abortion went into effect—these concerns were hypothetical, as there had yet to be any reported instances of these consequences materializing. Still, our clinicians took the risks very seriously. Those committed to continuing to share abortion information often expressed their belief that they were in peril, offering comments like, ‘some people just run into the fire’.^67^

In this section, we take the measure of the negative consequences that might follow for clinicians who share abortion information, beginning with the most serious of risks: the possibility that providing basic abortion information could lead to prosecution. A detailed analysis of the threat of criminal liability is beyond the scope of this paper, as each state has its own criminal code and relevant case law. Instead, we offer here some general thoughts on the risks of criminal liability for sharing abortion information.

#### IV.A.1. The Legality of Sharing Abortion Information

We begin by noting that, at least as of this writing, no state has a law that expressly makes it a crime to share abortion information. Such a law, if enacted, would face some headwinds, as it necessarily conflicts with current First Amendment doctrine by impinging on the clinician’s Constitutional right to free speech.^68^ In other contexts, courts have rejected state laws that attempt to stop doctors from sharing health information. For example, in *Wollschlaeger v. Governor of Florida*, the 11th Circuit Court of Appeals struck down a Florida statute barring doctors from sharing gun safety information, on the grounds that it constituted a free speech violation.^69^

It is useful, though, to imagine a ban on sharing abortion information, because such a law squarely presents the conflict that arises when a doctor’s ethical and professional obligations are at odds with the law, thereby calling into view the role of civil disobedience. Ethicists Dena Davis and Eric Kodish address this challenge in their 2014 essay about how doctors should respond when laws conflict with medical ethics: “In situations that pose a conflict between ethical conduct and abiding by an unjust law, an act of civil disobedience may be indicated. A doctor should commit civil disobedience rather than lie to a patient.”^70^ In their analysis, civil disobedience serves both to safeguard professional integrity and as a bulwark against an unjust law. As they suggest, “if all the affected doctors did this, the law would disappear very soon.”^71^

But in the absence of laws specifically outlawing the sharing of abortion information, it is vital to note that those who share it *are not* committing civil disobedience. Indeed, as medical ethicist Katie Watson argues in a slightly different context:

“[it] is neither civil disobedience nor covert lawbreaking; it isn’t even resistance. It is wise interpretation of existing law as applied to specific facts, fidelity to clinicians’ fiduciary duty to stay focused on patients in medical need, and acceptance that choices of historic consequence rarely come with zero risk.”^72^

#### IV.A.2. Doctors as Accomplices

Even without direct bans on sharing abortion information, doctors worry that sharing abortion information might implicate them as accomplices to abortion crimes committed by others—most notably, by their patients.^73^ The allegation here would be that for doctors practicing where abortion is banned, telling patients about options for accessing abortion makes them an accomplice in the event the patient goes on to have one. It was the risk of being charged as an accomplice that most troubled our interviewees. As one said, “[s]he tells her next-door neighbor, [and the] next-door neighbor makes sure that I’m on the hook for them.”^74^

In theory, accomplice liability poses a grave threat because even if they did not commit the criminal act itself, accomplices typically can be convicted of the crime they helped another to commit. That is to say, an accomplice to robbery is guilty of robbery.^75^ With various state laws making it a felony to perform an abortion, doctors are understandably concerned. But upon closer examination, any such prosecution will encounter multiple hurdles, making this fear largely unfounded.

To begin, note that in order to be an accomplice, the act one aids must itself be illegal. Yet for clinicians sharing abortion information in states that criminalize the provision of abortion, this basic condition may not be met. For example, there is no crime committed if a patient, acting on the information her doctor provides, later obtains an abortion from a provider practicing in a state where abortion is legal. She will have had a legal abortion.^76^ Although some state lawmakers have proposed making it a crime to cross state lines to have a legal abortion, the implications for federalism and the balance of states’ rights, not to mention individual rights, are so far-reaching that as of yet, no state has passed such a law.^77^

A related challenge arises in the event that the patient opts to self-manage an abortion, say by acquiring pills from information obtained via a website mentioned by their doctors. Here, too, there’s the challenge of identifying the criminal act, as most state laws criminalizing abortion do not impose criminal penalties on those who seek abortions. Although some anti-abortion state lawmakers advocate criminalizing self-managed abortion, to date, they have faced opposition from across the political spectrum from those concerned about the negative downstream implications of punishing those who have abortions.^78^

The risk of accomplice liability, then, requires us to assume that self-managed abortion is illegal, or that, in self-managing their abortion, a patient breaks other laws, for example, by importing abortion medication in violation of state law.^79^ The question then becomes whether the doctor who provided abortion information has ‘aided’ or ‘assisted’ that crime. To secure a conviction, the state must establish, beyond a reasonable doubt, each of the elements of the crime, beginning with the question of what the clinician intended, when supplying abortion information. In many jurisdictions, the prosecution must prove the alleged accomplice ‘intended’ for the perpetrator to commit the target crime.^80^ This is a hard standard to meet in the case of doctors who share abortion information, as their intention typically is not to encourage their patients to end their pregnancies, but rather, to promote patients’ well-being, enabling them make an informed decision, consistent with sound medical practice, as reflected in ethical and professional norms.

Other jurisdictions simply require that one provide assistance to someone, knowing they will break the law.^81^ Yet even under this easier legal standard, the prosecution will face two hurdles. First, they must establish beyond a reasonable doubt that the clinician ‘knew’ the patient intended to have an illegal abortion. This typically will be hard to establish, given that the information shared included legal options such as continuing the pregnancy and parenting or placing for adoption. Second, the state must prove that providing abortion information amounted to enough assistance (typically called ‘material assistance’) to implicate them in the underlying crime.

Ultimately, the question of how much assistance is required to be considered an accomplice will turn on the case law of any given jurisdiction. It is interesting, though, to consider how we understand doctors’ information-based assistance in other contexts. In response to the advent of state laws permitting medical marijuana, the federal government sought to restrict doctors’ licenses based on their having recommended medical marijuana to a patient.^82^ The case of *Conant v. Walters* involved a successful challenge to this policy, which the 9th Circuit Court of Appeals enjoined, noting that although the government retains the ability to sanction doctors who aid and abet the *actual* distribution and possession of marijuana, the sharing of information by doctors was protected by their First Amendment rights.^83^

Similarly, one might consider the question of information regarding medical assistance in dying. Although the practice is legal in some states, it remains illegal in others.^84^ There is little doubt that, were a doctor practicing in a state that outlaws medical assistance in dying to knowingly write a prescription for a lethal dose of medicine, that doctor would open themselves to criminal charges, including the possibility of being charged as an accomplice to a homicide. But it seems absurd to imagine the same charges applying to a doctor who supplies their patient with information about which states permit medical assistance in dying.

In addition to the challenges of proving a legal case against the doctor who provides abortion information, the prosecution will face equal or greater challenges in the court of public opinion. It remains to be seen whether judges, juries, and voters—district attorneys typically are elected officials—will support bringing charges against doctors, or rather, will resent the effort of prosecutors to muzzle doctors who were acting to safeguard their patients’ well-being and fulfilling their professional obligations.^85^ Polling shows that in all but six states, a solid majority of the public believes that abortion should be legal in all or most cases.^86^ Thus, there is good reason to believe that the prosecution of clinicians for sharing abortion information in the context of providing patient care would be unpopular.

For now, clinicians are practicing in the shadow of the law, struggling to gauge and limit their risks when sharing abortion information. The result is a classic illustration of the law’s chilling effect: because the laws are vague and the risks potentially serious, anti-abortion lawmakers have incentivized doctors to deprioritize their patients’ best interests and alter sound medical practice. Of course, that result accomplishes much of what abortion opponents want, without requiring them to pass controversial laws or bring unpopular prosecutions.

#### IV.A.3. Civil and Professional Risks

Even if the risks of being convicted of a crime for having shared abortion information are minimal, the reality is that, from the clinician’s perspective, being charged with a crime may be its own punishment, leading to reputational and personal harm and potentially weightier consequences.^87^ Then there’s the added risk of civil liability in states with bounty laws like Texas, in the form of lawsuits seeking monetary damages connected with aiding an abortion.^88^ Finally, there are a constellation of negative professional consequences—job loss, reputational damage, and loss of license—that might stem from being prosecuted or sued, or even being ‘outed’ as a provider of abortion information.^89^

Our interviewees were keenly aware of these risks and of the moral calculus they imposed upon individual providers. One clinician explained:

I think there will probably be two camps. Like there will be some people that will be like, I’m just going to run into the fire … I’m going to do what I can and I’m okay with it. And, you know, it’s good that we always have those people. But there are going to be other people that are just like, you know, … they have their whole career ahead of them. They’re in a lot of debt. They’re worried about losing a license, which is their whole way out of debt. … And I can’t blame them. Right? I mean, like, that is taking a huge risk and you’re already financially strapped. …You know, I mean, I’m 55 years old and I paid off my medical debt at age 50. So, that was how long it took me to pay off my med school. And ... I don’t have near the debt that people are graduating with, you know. And so like, there’s only so much that people can actually risk, and have a family, and pay their bills, and keep a roof over their head, you know?^90^

Many of our interviewees expressed frustration at the struggle to balance ethical provision of care with legal uncertainty. One stated:

To be honest with you, I’m at the point in my career, I want somebody to fucking sue me for this. I want to be arrested for guiding a patient [on how] to get an abortion. Don’t tell my wife that, but I would love for that to happen, for me to do my job, which is the right thing. I’m not performing the abortion, but I am taking care of my patients. I would love to be the one who gets arrested for that. I mean, honestly… it’s taking care of my patients and every organization in America would defend me, ACOG, the ACLU, you know, every professional organization would.^91^

These concerns have been stoked, rather than settled, by the climate of confusion and uncertainty that prevails when it comes to the permissibility of counseling patients about abortion options. Consider the likely impact on clinicians of Idaho attorney general Raúl Labrador’s threat, in March 2023, to suspend the licenses of providers on the grounds that Idaho law “prohibits an Idaho medical provider from … referring a woman across state lines to access abortion services.”^92^ After being sued for Constitutional violations by the ACLU and local physicians, he quickly withdrew his advisory opinion.^93^ Nonetheless, one can understand why the episode might make providers in Idaho and elsewhere hesitant to test the law by sharing abortion information.

There is a toll taken on clinicians asked to practice in this legal climate. Moreover, worries about the legal and professional risks of providing abortion information come at a time when clinicians already are struggling with the ongoing challenges and burdens growing out of the Covid pandemic. As one of our clinicians noted:

Like now we have to worry about people surreptitiously recording us and, you know, reporting us to the state and things that just drive people out of medicine. Like I think we’re already at the edge of a lot of burn-out with the pandemic and on top of that, you’re adding a lot more burden.^94^

The reality is that we are unlikely to see a quick resolution to the legal uncertainties that accompany the provision of abortion-related information. After all, because it incentivizes doctors to pull back from providing standard of care medicine, including all-options counseling, the uncertainty is working well for abortion opponents. But the other reality is that the medical profession is in a position to lessen the burdens being shouldered by clinicians in this uncertain environment.

### IV.B. Strategies for Safeguarding the Profession and Minimizing Risks to Clinicians

The chilling effect of abortion bans threatens not only patients’ well-being but also providers’ well-being. And, by undermining the doctor–patient relationship, the laws corrode the integrity of the medical profession as a whole. In the wake of the Dobbs decision, there have been profession-wide responses to criminalizing abortion. For example, the American Medical Association (AMA) issued a statement decrying the decision as:

[A]n egregious allowance of government intrusion into the medical examination room, a direct attack on the practice of medicine and the patient-physician relationship, and a brazen violation of patients' rights to evidence-based reproductive health services….^95^

In November 2022, the AMA reminded clinicians of their duty to follow ethical practice, even when it is illegal, referencing the preamble to the AMA Code of Ethics, which states, “When physicians believe a law violates ethical values or is unjust … ethical responsibilities should supersede legal duties.”^96^ At the same meeting, the AMA House of Delegates amended its Ethics Opinion on abortion to delete the phrase ‘under circumstances that do not violate the law’ in its description of when it is ethical to perform abortions.^97^

But clinicians need more than exhortations and defense lawyers to offset their concerns about providing abortion information. They need precise guidelines from their health care systems and professional organizations. Professional medical societies in any clinical practice specialty in which providers might encounter pregnant patients must unambiguously notify their members that the provision of abortion information is the standard of care. Clinicians must be informed that all-options counseling is not just “permissible” but required for any clinician who treats pregnant patients.

The groundwork for clinical practice directives already has been laid. In 2018, the AAFP, AAP, ACOG, and ACP issued a set of ‘Joint Principles Protecting the Patient-Physician Relationship: Keeping External Interference Out of the Practice of Medicine’, which calls on its members and policymakers to:

Reject government restrictions on the information our patients can receive from their doctors. Patients expect medically accurate, comprehensive information from their physicians; this dialogue is critical to ensuring the integrity of the patient-physician relationship. No governmental body should interfere in our members’ obligation to provide evidence-based information to their patients. When our government restricts the information that can be given to women, or forces physicians to provide women with non-medically inaccurate information, we can expect increased rates of unplanned pregnancy, pregnancy complications, and undiagnosed medical conditions.^98^

But to date, existing guidance from professional societies stops short of directing members to provide abortion information. For example, in June 2022, the AAP issued a policy statement providing that pediatricians should

(i) Inform the pregnant adolescent of all their options, which include continuing the pregnancy and raising the child; continuing the pregnancy and making an adoption, kinship care, or foster care plan; or terminating the pregnancy.
(ii) Be prepared to provide a pregnant adolescent with accurate information about each of these options in a developmentally appropriate manner involving a trusted adult, when possible; support the decision-making process; and assist in making connections with community resources that will provide quality services during and after the pregnancy.^99^

Yet the same declaration undercuts these provisions with the following statement:

“The AAP acknowledges the tension that pediatricians may face between their ethical duty to the patient and their duty to observe the law, and that pediatricians may choose not to follow these AAP recommendations when it is illegal to do so.”^100^

At face level, this statement doesn’t undercut the duty to provide abortion information because, as we have explained, there are no laws making it illegal for doctors to share abortion information. But let’s be honest: until a state supreme court rules that its abortion ban does not bar clinicians from sharing abortion information, there will be uncertainty about whether all-options counseling is legally risky. Hence, physicians will continue to worry about providing it. From this vantage, it is hard to read AAP’s caveat as anything other than blanket permission for its members to pull back from sound medical practice.

We understand why clinical practice specialty organizations might view such flexible guidance as a compassionate nod to the pressures leading clinicians to restrict their practices, but notice how, in making allowance for opting out of providing sound medical care, they remake the standard of medical care into a matter of personal choice. In the event of a prosecution, the doctor who opted to follow the aspirational guidelines is effectively stripped of the ability to justify their actions by reference to a professional baseline.

The more specific professional societies are about the obligation to provide abortion information, the better they will protect clinicians.^101^ Practically speaking, clinicians struggling to stay abreast of the changing abortion information landscape will be aided by knowing what abortion information they are expected and allowed to share. In principle, there is no one answer to this question, as the clinician’s obligation is tailored to the individual patient and reflects their duty to ensure that necessary health information is conveyed in a manner that their patient can understand. But there are some basic minimums that should easily make the list of required abortion information: the fact that abortion is legal in other states, the names of trustworthy organizations or websites that will help the patient understand their options, and explicit guidance about digital privacy.^102^

In addition to any local organizations, clinicians should share the names of organizations that will help keep their patients safe. One simple, yet comprehensive website is the Reprocare Healthline, an anonymous text and call line providing peer-based emotional support, medical information, and referrals to people having abortions.^103^ Additional options might include groups like “I Need an Abortion–ineedana.com,” AidAccess, Plan C, Women Help Women and the M + A Hotline. Professional societies might also consider drafting a scripted response, both to educate their membership and to protect clinicians by permitting them to defend themselves by referencing their professional guidelines.^104^ Professional societies and hospitals might also help clinicians and patients by creating information sheets for patients that share the above basic information about abortions.

To be sure, a professional society or hospital policy declaring that doctors must provide abortion information—that the failure to provide it constitutes negligence—will not prevent doctors from being sued or prosecuted. Nonetheless, it will make plain that prosecutors and bounty hunters are attacking not just the individual clinician but also the profession as a whole by endeavoring to use the law to compel doctors to betray both their vulnerable patients and their professional obligations.

A further benefit of clear professional guidelines is that they help shield clinicians from negative professional consequences such as job loss for having shared abortion information. In making it clear that sharing abortion information is the standard of care, the profession will make it harder for hospitals, medical malpractice underwriters, and state licensing boards to wreak havoc with the livelihood of individual clinicians.

These guidelines would go a long way toward offsetting the risk calculus that is driving the move away from robust all-options counseling. It bears remembering that, because uncertainty about risks has enabled abortion opponents to change the provision of abortion-related care without having to enforce unpopular laws, there is little reason to expect clarification to be forthcoming. In the meantime, clinicians rely on hospital counsel to guide them about what is legal, what is illegal, and what is risky. This reliance is inherently problematic, in that these lawyers may mistakenly understand their primary job as protecting their institutions from legal risk, rather than protecting patients from harm. It is unethical for an in-house counsel’s advice to ignore the relatively low legal risks of all-options counseling and their duty to zealously protect the rights and needs of those whom their institution exists to serve.^105^ But to the extent that the medical profession sends clear signals about the rights and needs of its providers and patients, its lawyers will more readily incorporate these vital factors into their legal advice.

Finally, the medical profession must not turn a blind eye to evidence that some doctors are failing to provide abortion information. Tolerating, let alone embracing, such behavior necessarily undermines the integrity of the entire profession. In addition to stepping up to defend clinicians who provide abortion information, the profession should proactively enforce community norms by censuring those who breach their duty by failing to do so. One of the most powerful ways to underscore the centrality of the duty to provide abortion information would be to censure those found to have breached it. Professional organizations such as the AMA, ACOG, and the AAP have codes of ethics, which their members are expected to follow.^106^ These organizations can formally censure or reprimand members who violate these standards, both by suspending or revoking membership and by referral to the appropriate state medical board for review.^107^ In view of the impact on patients’ well-being, alongside the threat to the profession as a whole, it would seem that hospitals and licensing boards have a far stronger set of justifications for censuring those who fail to provide abortion information than they do for those who do so.

## V. CONCLUSION

We are living in uncertain times, and there is no simple way to placate clinicians’ fears about the potential risks of providing abortion information. But doctors’ failure to share abortion information forces their patients to internalize risks that are at least as serious, and more likely to ensue. And because we are talking about patients, rather than strangers, doctors are duty bound to provide their patients with information that will permit them to navigate those risks.

In the end, the question of how doctors should respond when a patient needs abortion information, is not simply a legal or ethical matter. It is also a question of character, as is clear from the way one of our clinicians answered a question about whether they feared prosecution:

Oh, I worry about that all the time, you know? …I just, I guess really, honestly, I'm not sure what else to do except give people the information for them to make their decisions. And it's not like we're taking them there, …. We're just giving information so they can make the phone calls and if they want somebody sitting next to them, when they make the phone call, you know, then that's just kind, in my mind. … We, you know, we have to give hard things to people, [and] we just try to make sure that… they can identify a support person. [W]e don't care whether that's a peer or anything else, but we just want to know that we're not just saying ‘good luck with that one,’ you know, with no person that they could call about it. And sometimes the person they call is us and that's okay. Like I, I, I don't feel, and you know, maybe this is my being naive, but you know, a lot of what we're talking about is emotional support. Right? It's not, it's not even necessarily information. It's just… being with somebody when they got some news that was not what they were hoping for. And that feels different.^108^

## Footnotes

1 See Caron R. Kim et al., *Enabling Access to Quality Abortion Care: WHO’s Abortion Care Guideline,* 10 The Lancet 3467 (2022), https://www.thelancet.com/pdfs/journals/langlo/PIIS2214-109X(21)00552-0.pdf.

2 See Diana Lara et al., *Knowledge of Abortion Laws and Services Among Low-Income Women in Three United States Cities*, 17 J. Immigrant Minority Health 1811 (2015), https://pubmed.ncbi.nlm.nih.gov/25488893/. See also Adrianna Rodriguez, *Latinas have long been targeted my abortion misinformation. It’s getting worse, experts say.*, USA Today (Nov. 4, 2022), https://www.usatoday.com/story/news/health/2022/11/04/abortion-misinformation-latinas-roe-midterm-elections/8079815001/ (accessed Apr. 18, 2023).

3 See Grace Sparks, et al., *KFF Health Tracking Poll: Early 2023 Update on Public Awareness on Abortion and Emergency Contraception*, Kaiser Fam. Found. (Feb. 1, 2023), https://www.kff.org/womens-health-policy/poll-finding/kff-health-tracking-poll-early-2023/ (accessed Apr. 18, 2023) (finding evidence suggests that the majority of Americans are uncertain about the legality of medication abortion).

4 Regarding conscientious objectors, it is vital to note that clinicians who know they will refuse to provide abortion-related care should declare this position both to their patients and to their colleagues before, rather than after, they begin treating their patients, so as to ensure continuity of care, consistent with their ethical and professional obligations. See, eg American Academy of Pediatrics, *Options Counseling for the Pregnant Adolescents*, 150 Pediatrics 1 (2022), https://publications.aap.org/pediatrics/article/150/3/e2022058781/188340/Options-Counseling-for-the-Pregnant-Adolescent (“[Physicians should] examine their own beliefs and values to determine whether they can provide nonjudgmental, factual pregnancy options counseling that includes the full range of pregnancy options. If they cannot fulfill this role, they should facilitate a prompt referral for counseling by another knowledgeable professional in their practice setting or community who is willing to have such discussions with adolescent patients. The impact on the patient should be minimized and the patient should not know the reasons a referral to another provider is needed”).

5
*Dobbs v. Jackson Women’s Health Org.*, 142 S. Ct. 2228 (2022).

6 See *Institutional Review Boards (IRBs) and Protection of Human Subjects in Clinical Trials*, U.S. Food and Drug Administration, https://www.fda.gov/about-fda/center-drug-evaluation-and-research-cder/institutional-review-boards-irbs-and-protection-human-subjects-clinical-trials (accessed Apr. 19, 2023).

7 See *Certificates of Confidentiality*, Nat’l Inst. Health, https://grants.nih.gov/policy/humansubjects/coc.htm (accessed Apr. 19, 2023).

8 See *Abortion Rates by Race and Ethnicity*, Guttmacher Institute (Oct. 19, 2017), https://www.guttmacher.org/infographic/2017/abortion-rates-race-and-ethnicity (accessed Apr. 19, 2023).

9 Ten of our 25 participants worked in maternal–fetal medicine; nine worked at academic medical centers; and nine worked at religiously affiliated hospitals. Sixteen of our participants identified as female.

10 Mark S. Handcock & Krista J. Gile, *Comment: On the Concept of Snowball Sampling*, 41 Sociol. Methodol. 367 (2011), https://journals.sagepub.com/doi/10.1111/j.1467-9531.2011.01243.x (snowball sampling in sociology and statistical research is a recruitment technique of study where existing subjects are asked to identify future subjects from among their acquaintances).

11 See Interview with K (July 11, 2022) (on file with author) (“I would be petrified that [the Attorney General] would prosecute me. … Somebody is 10 weeks pregnant. They start bleeding, they start passing tissue. Okay. And I do an ultrasound on them and they’re bleeding heavily. I know they have tissue in the uterus. So I’d do a suction D&C. And again, I’d be scared to death right now that I was going to be sued. So these are things that have nothing to do with abortion, but it has to do with practicing obstetrics. Yeah. That puts a doctor in a position. Is [the AG] going to prosecute me?”)

12 Interview with W (July 18, 2022) (on file with author) (“I have a very good friend [from] residency… and she mentioned that the day after …our trigger laws went into effect, she had a 16-week patient. Water broke, leg hanging in the vagina, and she could not intervene because there was a heartbeat. She didn’t have a fever, elevated white cell count. She didn’t have anything else that she could hang her hat on. [W]e know this isn’t going to end well. This is not something that’s going to seal up. …And even though we all know that, … no physician wants to be the one that is the example. You know, that’s going to jail and losing their license and their ability to support their family and practice in their community.”)

13 See, eg Kate Zernike, *Five Women Sue Texas Over the State’s Abortion Ban*, N.Y. Times, Mar. 6, 2023, https://www.nytimes.com/2023/03/06/us/texas-abortion-ban-suit.html (accessed Apr. 13, 2023).

14 Many states require clinicians to provide patients seeking abortion with so-called ‘informed consent’ materials, which include medically contested and misleading assertions such as claims relating to fetal pain, and warnings that abortion causes breast cancer or depression. See eg *How Does Your State Compare*? Informed Consent Project, https://informedconsentproject.com/states/ (accessed Apr. 12, 2023). The American College of Obstetricians and Gynecologists (ACOG) objects to laws that ‘unduly regulates or criminalizes abortion care providers, including by…[f]orcing physicians to give patients inaccurate or biased information.’ It does not, however, require providers to dispel the inaccuracies by supplying accurate information. See *Policy Priorities: Abortion Access*, American College of Obstetrics and Gynecology, https://www.acog.org/advocacy/policy-priorities/abortion-access (accessed Apr. 12, 2023).

15 Interview with O (July 12, 2022) (on file with author) (“You’re required to give them this …informed consent booklet, which, I don’t know if other providers have kind of talked to you about that, but like, my head like exploded when I moved here and realized that I had to offer every patient a book that literally starts out: ‘[L]ife begins at conception. And when you perform an abortion, you kill like an individual single life.’ So I say things like, ‘Hello, this book is, you know, mandated by the state for me to offer you.’ It contains incorrect medical information in it. Crazy things like ‘abortion causes breast cancer,’ which is not true.”) See also Mara Buchbinder et al., *Reframing Conscientious Care: Providing Abortion Care When Law and Conscience Collide*, 46 Hastings Ctr. Rep. 22 (2016) (a compelling study documenting similar responses among doctors compelled by state law to share inaccurate abortion information).

16 Interview with N (July 12, 2022) (on file with author).

17 Interview with D (July 6, 2022) (on file with author).

18 Interview with B (July 6, 2022) (on file with author).

19 Interview with D (July 6, 2022) (on file with author).

20
*Id.*

21 We will be conducting a second round of interviews in Summer 2023 to test this hypothesis.

22 See *infra* Section IV: Confronting the Risks of Providing Abortion Information (arguing that the vagueness in laws criminalizing abortion is more a feature than a bug, permitting states criminalizing abortion to effectively limit access to abortion without requiring them to pass controversial laws or bring unpopular prosecutions).

23 Brittni Frederiksen, Usha Ranji, Ivette Gomez, Alina Salganicoff, *A National Survey of OBGYNs’ Experiences After Dobbs*. Kaiser Family Foundation, June 21, 2023. https://www.kff.org/report-section/a-national-survey-of-obgyns-experiences-after-dobbs-report/ (accessed July 8, 2023).

24 Interview with D (July 6, 2022) (on file with author) (“I mean, I got a wife and two kids, I don’t really want to spend the next 10 years of my life wondering if I’m going to get to go before the Supreme Court or to prison, because in the meantime I have to like practice medicine to feed the family.”)

25 Caron R. Kim et al., *Enabling access to quality abortion care: WHO’s Abortion Care Guideline,* 10 Lancet Glob. Health e467 (Apr. 2022), https://www.thelancet.com/pdfs/journals/langlo/PIIS2214-109X(21)00552-0.pdf.

26 See, eg *Facts Are Important: Abortion Is Healthcare*, American College of Gynecology and Obstetricians, https://www.acog.org/advocacy/facts-are-important/abortion-is-healthcare (accessed Apr. 12, 2023); *Preserving Access to Reproductive Health Services D-5.999*, American Medical Association, https://policysearch.ama-assn.org/policyfinder/detail/abortion?uri=%2FAMADoc%2Fdirectives.xml-D-5.999.xml (accessed Apr. 13, 2023) (“Our AMA: (1) recognizes that healthcare, including reproductive health services like contraception and abortion, is a human right …”); *Letter in Support of U.S. Dep’t of Veterans Aff.s’ proposal to amend its medical regulations Re: Reproductive Health Services*, American College of Emergency Physicians, Oct. 11, 2022, https://www.acep.org/siteassets/new-pdfs/advocacy/acep-response-to-va-reproductive-health-ifr-10.11.22.pdf (accessed Apr. 13, 2023) (“It is important for pregnant veterans and [Civilian Health and Medical Program of the Department of Veterans Affairs] beneficiaries in medical emergencies to understand and be aware of all medically appropriate treatment options and their implications, including pregnancy termination”); *An Update to ACP’s Women’s Health Policy in the United States*, American College of Physicians, May 23, 2022, https://assets.acponline.org/acp_policy/policies/updated_womens_health_policy_position_statement_2022.pdf (accessed Apr. 13, 2023) (“ACP believes that individuals have the right to make their own decisions, in partnership with their physician or health care professional, on matters affecting their individual reproductive health and opposes government restrictions that would erode or abrogate one’s right to continue or discontinue a pregnancy that may result from the Supreme Court ruling in Dobbs v. Jackson Women’s Health Organization.”). See also, Brief of Amici Curiae in Support of Plaintiff’s Motion for a Preliminary Injunction, *United States v. State of Idaho*, No. 1:22-cv-00329-BLW (D. Idaho Aug. 24, 2022) (amici included American College of Emergency Physicians (ACEP), American College of Obstetricians and Gynecologists (ACOG), American Medical Association (AMA), Society for Maternal-Fetal Medicine, National Medical Association, National Hispanic Medical Association, American Academy of Pediatrics, American Academy of Family Physicians, and American Public Health Association).

27
*Facts Are Important: Abortion Is Healthcare*, The American College of Obstetrics & Gynecologists, https://www.acog.org/advocacy/facts-are-important/abortion-is-healthcare (accessed Apr. 12, 2023).

28 See, eg Lisa B. Haddad and Nawal M. Nour, *Unsafe Abortion: Unnecessary Maternal Mortality*, 2 Rev. Obstet. Gynecol. (2009) (discussing the relationship between rates of unsafe abortion and restrictive abortion laws). See also, Dovile Vilda et al., *State Abortion Policies and Maternal Death in the United States, 2015-2018*, 111 Am. J. Pub. Health 1696 (2021), https://pubmed.ncbi.nlm.nih.gov/34410825/; Roman Pabayo et al., *Laws Restricting Access to Abortion Services and Infant Mortality Risk in the United States*, 17 Int’l J. Env’t Rsch. Pub. Health 3773 (2020) (discussing the relationship between the type and number of state-level restrictive abortion laws and infant mortality risk); Sherajum Monira Farin et al., *The Impact of Legal Abortion on Maternal Mortality*, Sept. 1, 2021, https://papers.ssrn.com/sol3/papers.cfm?abstract_id=3913899 (accessed Apr. 19, 2023).

29 See, eg Abigail R.A. Aiken et al., *Safety and effectiveness of self-managed medication abortion provided using online telemedicine in the United States: A population-based study*, 10 Lancet 100,200 (June 2022), https://doi.org/10.1016/j.lana.2022.100200.

30 See *Abortion: Access and Safety Worldwide*, 391 The Lancet 1121 (Mar. 24, 2018), https://www.thelancet.com/journals/lancet/article/PIIS0140-6736(18)30624-X/fulltext. See also, Susheela Singh et al., *Abortion Worldwide 2017: Uneven Progress and Unequal Access*, Guttmacher Institute, March 2018, https://www.guttmacher.org/report/abortion-worldwide-2017;  *Unsafe abortion incidence and mortality: Global and regional levels in 2008 and trends during 1990-2008*, World Health Org., 2012, https://apps.who.int/iris/bitstream/handle/10665/75173/WHO_RHR_12.01_eng.pdf (accessed Apr. 13, 2023). See also Aaron Nelson, *Taking Calls on Abortion, and Risks, in Chile*, N.Y. Times, Jan. 3, 2012, https://www.nytimes.com/2013/01/04/world/americas/in-chile-abortion-hot-line-is-in-legal-gray-area.html (accessed Apr. 13, 2023) (describing the establishment of abortion hotlines to provide information to men and women seeking abortion in South American countries where abortion is illegal).

31 See J. Sherris et al., *Misoprostol Use in Developing Countries: Results from a Multi-country Study*, 88 Int’l J. Gynecol. Obstet. 76, 77 (2005).

32
*WHO issues new guidelines on abortion to help countries deliver lifesaving care*, World Health Org. (Mar. 9, 2022), https://www.who.int/news/item/09-03-2022-access-to-safe-abortion-critical-for-health-of-women-and-girls (accessed Apr. 12, 2023).

33 See, eg Elizabeth G. Raymond and David A. Grimes, *The comparative safety of legal induced abortion and childbirth in the United States*, 119 Obstet. Gynecol. 215 (2012), https://pubmed.ncbi.nlm.nih.gov/22270271/.

34 See *infra* notes 49-53 and accompanying text.

35 See *Open Door Counseling and Dublin Well Woman v. Ireland*, 246 Eur. Ct. H. R. (ser. A) (1992), https://www.globalhealthrights.org/wp-content/uploads/2013/02/ECtHR-1992-Open-Door-and-Dublin-Well-Woman-v.-Ireland.pdf. The USA is not a member nation of the European Court of Human Rights, and so is not bound by its decisions, but the ruling indicates the norms of human rights law as interpreted by other Western democracies.

36
*Id.*, at 25.

37 See Joanna N. Erdman, *Access to Information on Safe Abortion: A Harm Reduction and Human Rights Approach*, 34 Harv. J.L. Gender 413 (2011) (making a case for promoting access to abortion information both as a matter of human rights and sound public health policy).

38
Tom L. Beauchamp & James F. Childress, Principles of Biomedical Ethics (8th ed. 2019).

39
*Id.*

40 See Beauchamp & Childress, *supra* note 38.

41 See Nancy D. Berkman et al., *Low Health Literacy and Health Outcomes: An Updated Systematic Review*, 155 Ann. Intern. Med. 97 (2011).

42 See Kristine Sørensen et al., *Exploring the Ethical Scope of Health Literacy – A Critical Literature Review*, 2 Albanian Med. J. 70 (2013).

43 See Laura Huss, Farah Diaz-Tello, & Goleen Samari, *Self-Care, Criminalized: August 2022 Preliminary Findings*, If/When/How (2022), https://www.ifwhenhow.org/resources/self-care-criminalized-preliminary-findings.

44 See Beauchamp & Childress, *supra* note 39.

45
*Id.*

46 See *Healthy People 2030 Framework*, U.S. Dep’t. Health Hum. Res., https://health.gov/healthypeople/about/healthy-people-2030-framework (accessed Apr. 13, 2023).

47 See Nancy D. Berkman et al., *Low health literacy and health outcomes: an updated systematic review*, 155 Ann. Internal Med. 97 (2011), https://pubmed.ncbi.nlm.nih.gov/21768583/.

48 See Heather D. Boonstra, *Abortion in the Lives of Women Struggling Financially: Why Insurance Coverage Matters*, Guttmacher Institute (July 13, 2016) https://www.guttmacher.org/gpr/2016/07/abortion-lives-women-struggling-financially-why-insurance-coverage-matters.

49 Prior to Dobbs, 75 per cent of abortions went to people living below or just above the poverty line. See *Abortion rates by income*, Guttmacher Institute, Oct. 19, 2017, https://www.guttmacher.org/infographic/2017/abortion-rates-income (accessed Apr. 13, 2023). See also, Rachel K. Jones and Jenna Jerman, *Population Group Abortion Rates and Lifetime Incidence of Abortion: United States, 2008-2014*, 112 Am. J. Pub. Health 1284 (2022), https://ajph.aphapublications.org/doi/pdf/10.2105/AJPH.2017.304042.

50 See Diana Greene Foster et al., *Socioeconomic Outcomes of Women Who Receive and Women Who Are Denied Wanted Abortions in the United States,* 108 Am. J. Pub. Health 407 (2018), https://www.ncbi.nlm.nih.gov/pmc/articles/PMC5803812/.

51 See American Academy of Pediatrics, *Poverty and Child Health in the United States*, 137 Pediatrics 4 (2016). https://publications.aap.org/pediatrics/article/137/4/e20160339/81482/Poverty-and-Child-Health-in-the-United-States.

52 See *Joint Principles for Protecting the Patient-Physician Relationship*, American Academy of Family Practitioners, American Academy of Pediatrics, American College of Obstetricians and Gynecologists, American College of Physicians, May 23, 2018, https://www.groupof6.org/dam/AAFP/documents/advocacy/legal/ST-Group6-LegislativeInterference-052318.pdf.

53 See *Abortion Policy*, American College of Obstetricians and Gynecologists, https://www.acog.org/clinical-information/policy-and-position-statements/statements-of-policy/2022/abortion-policy (accessed Apr. 13, 2023).

54 See *Cancer Care and Reproductive Health*, Leuk. Lymphoma Soc’y, Association for Clinical Oncology, https://www.fightcancer.org/sites/default/files/cancer-care-and-reproductive-health-v4.pdf (accessed Apr. 13, 2023).

55 See *2022 Council Resolution 25: Advocacy for Safe Access to Full Spectrum Pregnancy Related Health Care*, American College of Emergency Physicians, https://webapps.acep.org/shoppingcart/printreport.aspx?vw=council&councilcode=SA22&resolutionnumber=25 (accessed Apr. 13, 2023).

56 See *World Medical Association Code of Medical Ethics*, World Med. Ass’n, https://www.wma.net/policies-post/wma-international-code-of-medical-ethics/ (accessed Apr. 13, 2023) (forbidding a doctor from allowing extraneous factors to corrupt sound medical practice and stating “[p]hysicians must take responsibility for their individual medical decisions and must not alter their sound professional medical judgements on the basis of instructions contrary to medical considerations.”

57 See Lynn M. Paltrow and Jeanne Flavin, *Arrests of and Forced Interventions on Pregnant Women in the United States, 1973-2005: Implications for Women’s Legal Status and Public Health*, 38 J. Health Pol. Pol’y REV. 299 (2013); *Arrests and Prosecutions of Pregnant People, 1973-2020*, Pregnancy Justice, Sept. 18, 2021, https://www.pregnancyjusticeus.org/arrests-and-prosecutions-of-pregnant-women-1973-2020/ (building on Lynn Paltrow and Jeanne Flavin’s study documenting arrests and detentions between 1973 and 2005) (accessed Apr. 19, 2023). See also *Decriminalizing Self-Managed and Supported Non-Clinical Abortion*, If/When/How, https://www.ifwhenhow.org/get-involved/strategic-initiatives-2/strategic-initiative-sma-may-2019-update/ (accessed Apr. 202,023); Laura Huss, Farah Diaz-Tello, & Goleen Samari, *Self-Care, Criminalized: August 2022 Preliminary Findings*, If/When/How (2022), https://www.ifwhenhow.org/resources/self-care-criminalized-preliminary-findings/ (discussing findings from research, 2000–2020) (accessed Apr. 20, 2023); Laura Huss, *Self-Managed Abortion is Not Illegal in Most of the Country, but Criminalization Happens Anyway*, If/When/How (Aug. 9, 2022), https://www.ifwhenhow.org/abortion-criminalization-new-research/ (providing findings from a multi-year research project to understand who has been targeted by criminalization for self-managing their abortion) (accessed Apr. 20, 2023); *Arrests and Prosecutions of Pregnant Women, 1973-2020*, Nat’l Advoc. for Pregnant Women (Sept. 18, 2021), https://www.nationaladvocatesforpregnantwomen.org/arrests-and-prosecutions-of-pregnant-women-1973-2020 (documenting race and class bias in the criminalization of behaviors alleged to pose risk to fetuses). See also Lynn M. Paltrow & Jeanne Flavin, *Arrests of and Forced Interventions on Pregnant Women in the United States, 1973-2005: Implications for Women’s Legal Status and Public Health*, 38 J. Health Pol. Pol’y Rev. 299, 304-05 (2013) (discussing these findings and the limitations of the research that led the authors to conclude that their findings represent a substantial undercount of cases). See Priscilla Thompson & Alexandra Turcios Cruz, *How an Oklahoma Women’s Miscarriage Put a Spotlight on Racial Disparities in Prosecutions*, NBC News (Nov. 5, 2021), https://www.nbcnews.com/news/us-news/woman-prosecuted-miscarriage-highlights-racial-disparity-similar-cases-rcna4583. See also Michele Oberman, Her Body, Our Laws: On the Frontlines of the Abortion War, From El Salvador to Oklahoma 43-67 (2018) (discussing how reports from doctors to police in El Salvador overwhelmingly involve poor, marginalized women). See generally Michele Goodwin, Policing the Womb: Invisible Women and the Criminalization of Motherhood (2020).

58 See *Arrests and Prosecutions*, Pregnancy Justice, *supra* note 59.

59 See Lynn M. Paltrow and Jeanne Flavin, *Arrests of and Forced Interventions*, at 313 (noting that the Black defendants were also significantly more likely to be charged with felonies than white women, with 85% of Black women receiving felony charges compared to 71% of white women). See also Lynn M. Paltrow, *Roe v. Wade and the New Jane Crow: Reproductive Rights in the Age of Mass Incarceration*, 103 Am. J. Pub. Health 17, 19 (2013). Note that health care experts object strenuously to these prosecutions on the grounds that they deter people from seeking treatment essential both to their own welfare and to that of the fetus. See eg Katherine C. Arnold, *Viewpoint: Criminalizing Young Women Is not the Way to Improve Birth Outcomes*, The Oklahoman (Dec. 26, 2021), https://www.oklahoman.com/story/opinion/2021/12/26/viewpoint-prosecuting-oklahoma-women-who-miscarry-wrong/8930865002/ (accessed Apr. 19, 2023).

60 See Michelle Oberman, Her Body, Our Laws, *supra* note 57 at 43-67 (2018). See also Jamila Perritt, *#WhiteCoatsForBlackLives: Addressing Physicians’ Complicity in Criminalizing Communities*, 383 N. Engl. J. Med. 1804 (2020).

61 In April 2022, Lizelle Herrera was arrested and charged with murder after a hospital notified police that she had sought care for complications from a self-managed abortion. See Caroline Kitchener et al., *A call, a text, an apology: How an abortion arrest shook up a Texas town*, Wash. Post, Apr. 13, 2022, https://www.washingtonpost.com/nation/2022/04/13/texas-abortion-arrest/ (accessed Apr. 14, 2023).

62 See *Texas prosecutor drops murder charge against woman arrested for self-induced abortion*, CBS News, Apr. 10, 2022, https://www.cbsnews.com/amp/news/lizelle-herrera-abortion-texas-murder-charge-dropped/ (accessed Apr. 14, 2023).

63 See, eg *Medical and Public Health Group Statements Opposing Prosecution and Punishment of Pregnant Women*, Pregnancy Justice, June 2021, https://www.pregnancyjusticeus.org/wp-content/uploads/2023/03/Medical-and-Public-Health-Group-Statements-Opposing-Prosecution-and-Punishment-of-Pregnant-Women.pdf (accessed Apr. 14, 2023). See also, *2022 Council Resolution 25*, American College of Emergency Physicians, *supra* note 55 (“ACEP opposes the criminalization or mandatory reporting for non-public health monitoring reasons of self-induced abortion as it increases patients’ medical risks and deters patients from seeking medically necessary services”); Yesenia M. Perez, *Ferguson v. City of Charleston and Criminalizing Drug Use During Pregnancy*, 15 AMA J. Ethics 771 (2013); American Medical Association Bd. Tr., *Legal interventions during pregnancy: court-ordered medical treatments and legal penalties for potentially harmful behavior by pregnant women*, 264 JAMA 2663 (1990), https://pubmed.ncbi.nlm.nih.gov/2098015/. (https://www.acep.org/what-we-believe/actions-on-council-resolutions/councilresolution/?rid=33B5D909-A225-ED11-A9D3-F94EF5641D50).

64 For further analysis and assessment of the legal risks of sharing abortion information, see Katie Watson and Michelle Oberman, *Abortion Counseling, Accomplice Liability, and the First Amendment*, N. Engl. J. Med. (2023) (forthcoming).

65 We are deeply indebted to Professor Katie Watson for conversation and thoughtful insight on the question of legal risks faced by doctors who share abortion information.

66 Interview with P (July 13, 2022) (on file with author).

67 Interview with L (July 11, 2022) (on file with author). Another participant invoked the same metaphor. Interview with R (July 14, 2022) (on file with author) (“We focus on the patient and kind of like put ourselves in everything else like second. … We run to the fire. But then now it’s almost like people are like, what, what do I put I do? It’s like illegal!”).

68 U.S. Const. amend. I. See also Sonia M. Suter, *The First Amendment and Physician Speech in Reproductive Decision Making*, 43 J. Law, Med. Ethics 22 (2015).

69 See, eg *Wollschlaeger v. Governor of Florida*, 848 F.3d 1293 (11th Cir. 2017). Nonetheless, with model legislation from the National Right to Life proposing to criminalize providing pregnant patients with information about self-managed abortion, it is perhaps only a matter of time before states attempt to make it a crime to share abortion information. See *Post-Roe Model Abortion Law*, Nat’l Rt. to Life, June 15, 2022, https://www.nrlc.org/wp-content/uploads/NRLC-Post-Roe-Model-Abortion-Law-FINAL-1.pdf. See also Veronica Stracqualursi, *National Right to Life eyes medication abortion restrictions as next step in post-Roe fight*, CNN, June 27, 2022, https://edition.cnn.com/2022/06/27/politics/national-right-to-life-convention-medication-abortion/index.html (accessed Apr. 14, 2023). In February 2023, a Texas lawmaker introduced a bill that would force internet providers to block access to any website that carries information about abortion medication or tells women how to get an abortion. See H.B. 2690, 88th Leg., Reg. Sess. (Tx. 2023), https://capitol.texas.gov/tlodocs/88R/billtext/pdf/HB02690I.pdf#navpanes=0) (accessed Apr. 14, 2023).

70 Dena Davis & Eric Kodish, *Laws that Conflict with the Ethics of Medicine: What Should Doctors Do*?, 44 Hastings Ctr Rep. 11, 13 (2014).

71
*Id.*

72 Katie Watson, *Dark-Alley Ethics: How to Interpret Medical Exceptions to Bans on Abortion Provision*, 388 N. Engl. J. Med. 1240 (2023).

73 Interview with I (July 8, 2022) (on file with author).

74
*Id.*

75 Every state has its own accomplice liability statute, but broadly speaking, an accomplice is someone who did anything to encourage, aid, or assist in any material manner in the commission of a crime – someone who “in some sort associate[s] himself with the venture, that he participate[s] in it as something that he wishes to bring about, that he seek[s] by his action to make it succeed.” *State of West Virginia v. Hoselton*, 179 W.Va. 645, 648 (W. Va. 1988) (quoting Learned Hand in *U.S. v. Peoni*, 100 F.2d 401, 402 (2nd Cir.1938)).

76 This issue is arising in the context of a wrongful death prosecution in Texas, in which defendants are charged for having conspired to kill a ‘human being’ after advising a friend about how to acquire abortion medication. See, eg Dahlia Lithwick and Mark Joseph Stern, *Sued for Offering Friendship*, Slate, Mar. 15, 2023, https://slate.com/news-and-politics/2023/03/texas-lawsuit-suing-friends-explained.html (accessed Apr. 14, 2023) (“Texas law expressly states that an individual does *not* commit a criminal act when she terminates her own pregnancy. The state’s abortion bans, homicide statute, and assault statute all declare that self-managed abortion is not a criminal act and cannot be punished as one. So even if Doe’s fetus ‘died’, for purposes of Texas law, its death was not ‘wrongful’, so no one can be held liable for abetting it. As Joanna Grossman, a visiting professor at Stanford Law School, told Slate, ‘If there’s no wrongful death, then there’s no wrongful death liability.’”).

77 A Missouri lawmaker, Mary Sue Coleman, proposed criminalizing traveling across state lines to obtain a legal abortion, but to date, no state has enacted such a law. For a rich, balanced consideration of the federalism challenges in barring residents from states with abortion bans from traveling to legal states in order to obtain abortions, see Susan Appleton (SSRN, forthcoming 2023). See also, Katherine Florey, *Dobbs and the Civil Dimension of Extraterritorial Abortion Regulation*, N.Y.U. L. Rev. (forthcoming 2023) (discussing civil remedies as an alternative or supplement to the criminal prosecution of out-of-state abortions and why current choice of law is not well equipped to resolve abortion issues in the coming civil litigation).

78 Caroline Kitchener, *Conservatives complain abortion bans not enforced, want jail time*, The Washington Post (Dec. 14, 2022) https://www.washingtonpost.com/politics/2022/12/14/abortion-pills-bans-dobbs-roe/.; Shefali Luthra, *Abortion Bans Don’t Prosecute Pregnant People. That May be About to Change.*, The 19th (Jan. 13, 2023) https://19thnews.org/2023/01/abortion-bans-pregnant-people-prosecution/.

79 Here, too, it bears noting that drug trafficking laws have yet to be applied to those who self-manage abortion, but a state might criminalize the possession or ingestion of abortion medication within the state, and, provided it can prove the patient acquired the drugs pursuant to information supplied by their doctor, the State might argue that the doctor aided and abetted their criminal act. See, eg Greer Donely and Jill Wieber Lens, *Abortion, Pregnancy Loss, and Subjective Fetal Personhood*, 75 Vanderbilt L. Rev. 1649, 1706 (2022) (“If states want effective abortion restrictions without a self-management loophole, they will most likely criminalize those who have abortions and the in-state residents who help them.”); David S. Cohen et al., *The New Abortion Battleground*, 123 Columbia L. Rev. 1, 19 (2023) (“Historically, abortion bans have targeted providers, but the rise of telehealth and self-management, where the provider might be beyond the state’s reach or nonexistence, suggests that enforcement of state abortion laws will target the people who seek abortion or those who assist them.”).

80 See Wayne R. La Fave, Criminal Law 892-93 (6th ed. 2017).

81
*Id.*

82
*Conant v. Walters*, 309 F.3d 629 (9th Cir. 2002) (describing federal marijuana policy promulgated in 1996 whereby the Department of Justice and the Department of Health and Human Services “cautioned that physicians who ‘intentionally provide their patients with oral or written statements in order to enable them to obtain controlled substances in violation of federal law’ ... risk revocation of their DEA prescription authority.”).

83
*Id*.

84
*Physician-Assisted Suicide Fast Facts*, CNN (May 26, 2022) https://edition.cnn.com/2014/11/26/us/physician-assisted-suicide-fast-facts/index.html.

85 Polls consistently find a majority of Americans oppose criminalizing abortion. See, eg *Abortion Attitudes in a Post-Roe World: Findings From the 50-State 2022 American Values Atlas*, Public Religion Research Institute, Feb. 23, 2023, https://www.prri.org/research/abortion-attitudes-in-a-post-roe-world-findings-from-the-50-state-2022-american-values-atlas/ (accessed Apr. 14, 2023). A January 2023 poll found that a majority of Americans worry that the threat of prosecution may deter clinicians from performing life-saving abortions. See Katherine Gilyard*, A vast majority of Americans are concerned people could face criminal penalties for abortion*, The 19th, Jan. 30, 2023, https://19thnews.org/2023/01/americans-concerned-possibility-abortion-crime/ (accessed Apr. 14, 2023).

86 Even in the six states where this was a minority position, it was a close call. In no case did less than 43 per cent of the population support legalized abortion. See *Abortion Attitudes*, Public Religion Research Institute, *id.*

87 For years, experts have noted the negative impact of medical malpractice litigation on clinicians’ mental health. See, eg Jerome W. Bettman Sr., *The Psychological and Emotional Impact of Being Sued*, Ophthalmic Mut. Insurance Co. Digest, 1993, https://www.omic.com/the-psychological-and-emotional-impact-of-being-sued/#:∼:text=The%20Medical%20Malpractice%20Stress%20Syndrome&text=These%20symptoms%20include%3A%20anger%2C%20inner,self%2D%20confidence%20and%20decreased%20libido, (accessed Apr. 14, 2023) (describing ‘medical malpractice stress syndrome’). See also Sara C. Charles, *Coping with a medical malpractice suit*, 174 West J. Med. 55 (2001), https://www.ncbi.nlm.nih.gov/pmc/articles/PMC1071237/ (accessed Apr. 14, 2023) (describing the emotional disequilibrium that accompanies being sued).

88 See S.B. 8, 87th Leg., Reg. Sess. (Tx. 2021). As of now, S.B. 8 has yet to generate much litigation, but by incentivizing private citizens to bring civil lawsuits against anyone suspected of aiding and abetting a prohibited abortion, there is every reason to believe we will see such lawsuits. In October 2021, three separate lawsuits were filed against Dr Alan Braid, a San Antonio-based physician, after he wrote an op-ed in The Washington Post revealing that he had performed an abortion in violation of the Texas Act. Two of the cases were never formally served, but the one filed by the Illinois resident did proceed through the courts. That case was dismissed on December 8, 2022, constituting the only S.B. 8 ruling to be resolved in court to date. The judge determined that the Illinois resident, as a bystander not directly impacted by the abortion, had no standing. See Madlin Mekelburg, *Texas Doctor Who Violated Abortion Law Wins Dismissal of Suit*, Bloomberg Law, Dec. 8, 2022, https://www.bloomberg.com/news/articles/ 2022-12-08/texas-doctor-who-violated-abortion-law-wins-dismissal-of-suit?leadSource=uverify%20wall (last accessed Dec. 14, 2022). See also Eleanor Klibanoff, *Anti-abortion lawyers target those funding the procedure for potential lawsuits under new Texas law*, Texas Tribune, Feb. 23, 2023, https://www.texastribune.org/2022/02/23/texas-abortion-sb8-lawsuits/ (accessed Apr. 14, 2023).

89 Although we are not aware of any clinicians who have been ‘outed’ or experienced retaliatory actions after counseling patients about abortion, abortion opponents have mounted personal attacks against those who have done so in other contexts. See eg Andrea González-Ramírez, *The Holy War Against One Pro-Abortion Rights Professor*, The Cut, April 3, 2023 (https://www.thecut.com/2023/04/tamara-kay-notre-dame-abortion-rights.html (accessed July 9, 2023) (describing the harassment of Notre Dame Professor Tamara Kay after she offered to share abortion information with students).

90 Interview with L (July 11, 2022) (on file with author).

91 Interview with K (July 11, 2022) (on file with author).

92 See, Raul L. Labrador, *Letter Re. Request for AG Analysis*, Mar. 27, 2023, https://759dc218-b8c7-48ed-a4df-e92eb29273e5.filesusr.com/ugd/be9708_de4a35f4a6854c0690cf88ecc810f97a.pdf (accessed Apr. 16, 2023).

93 See, eg Devan Cole, *Idaho AG rescinds legal opinion that said health care providers can’t make out of state abortion referrals*, CNN, Apr. 7, 2023, https://www.cnn.com/2023/04/07/politics/idaho-abortion-referrals-guidance-rescinded/index.html (accessed Apr. 16, 2023).

94 Interview with N (July 12, 2022) (on file with author). These concerns already are causing doctors and allied health professionals to leave the practice of medicine, at least in jurisdictions that criminalize abortion. See, eg Sophie Novack, *You Know What? I’m Not Doing This Anymore*, Slate, Mar. 21, 2023, https://slate.com/news-and-politics/2023/03/texas-abortion-law-doctors-nurses-care-supreme-court.html?utm_source=substack&utm_medium=emai (accessed Apr. 13, 2023).

95 See *Ruling an Egregious Allowance of Government Intrusion into Medicine*, American Medical Association, June 24, 2022, https://www.ama-assn.org/press-center/press-releases/ruling-egregious-allowance-government-intrusion-medicine (accessed Apr. 17, 2023).

96 See *AMA Announces New Adopted Policies Related to Reproductive Health Care*, American Medical Association, Nov. 16, 2022, https://www.ama-assn.org/press-center/press-releases/ama-announces-new-adopted-policies-related-reproductive-health-care (accessed Apr. 17, 2023).

97
Report of Reference Committee on Amendments to Constitution and Bylaws, American Medical Association House of Delegates (2022) (Interim meeting (I-22), Item 6, at 7). (https://www.ama-assn.org/system/files/i-22-refcomm-conby-report.pdf) (accessed July 9, 2023).

98 See American Academy of Family Practitioners, American Academy of Pediatricians, American College of Obstetricians and Gynecologists, American College of Physicians, *Joint Principles Protecting the Patient-Physician Relationship*, May 23, 2018, https://www.acponline.org/acp-newsroom/joint-principles-for-protecting-the-patient-physician-relationship (accessed Apr. 20, 2023).

99 American Academy of Pediatrics, *Options Counseling for the Pregnant Adolescents*, 150 Pediatrics 1 (2022), https://publications.aap.org/pediatrics/article/150/3/e2022058781/188340/Options-Counseling-for-the-Pregnant-Adolescent.

100
*Id.*

101
*Id.* We understand that conscientious objectors may be exempted from this requirement. As noted, they fall outside of the scope of our analysis (*see supra* note 5 and accompanying text). We note with approval the approach suggested by the American Academy of Pediatricians: “Pediatricians should… [e]xamine their own beliefs and values to determine whether they can provide nonjudgmental, factual pregnancy options counseling that includes the full range of pregnancy options. If they cannot fulfill this role, they should facilitate a prompt referral for counseling by another knowledgeable professional in their practice setting or community who is willing to have such discussions with adolescent patients.” See American Academy of Pediatricians, *Options Counseling supra* note 97.

102 Patients should be advised to use public computers, rather than personal devices, when searching for abortion information. They should likewise be advised not to text or post or share on social media any information about their pregnancy.

103 See Reprocare, https://abortionhotline.org/ (“Reprocare is a reproductive justice organization that seeks to holistically support access to abortion care in ways that confront economic and racial injustice.”). See also, eg Indeedana, https://www.ineedana.com/es/sobre-nosotrxs (“Nuestra meta es proporcionar una fuente localizada de información sencilla y al día para quienes buscan abortar” [trans.: “Our goal is to provide a simple, up-to-date, and localized source of information for people seeking abortions.”]); Abortion Finder, https://www.abortionfinder.org/ (“Abortion Finder is an easy-to-use search tool built on a database of over 750 verified abortion providers across the country.”).

104 We are not suggesting that doctors be required to deliver a specific ‘informed consent’ speech, but it bears noting that such a script would mark an interesting counterpoint to the scripted anti-abortion informed consent required by many states. See *supra* notes 15–16 and accompanying text.

105 See Model Rules of Professional Conduct, Rule 1.1 (2018). (“A lawyer shall provide competent representation to a client. Competent representation requires the legal knowledge, skill, thoroughness and preparation reasonably necessary for the representation.”), and Rule 2.1 (2021) (“In representing a client, a lawyer shall exercise independent professional judgment and render candid advice. In rendering advice, a lawyer may refer not only to law but to other considerations such as moral, economic, social and political factors, that may be relevant to the client’s situation.”).

106 See, eg *Code of Medical Ethics*, American Medical Association, https://code-medical-ethics.ama-assn.org/ (accessed Apr. 14, 2023); *Code of Professional Ethics*, American College of Obstetricians and Gynecologists, https://www.acog.org/-/media/project/acog/acogorg/files/pdfs/acog-policies/code-of-professional-ethics-of-the-american-college-of-obstetricians-and-gynecologists.pdf (accessed Apr. 14, 2023); Code of Conduct, American Academy of Pediatricians, https://www.aap.org/en/about-the-aap/american-academy-of-pediatrics-equity-and-inclusion-efforts/code-of-conduct/ (accessed Apr. 19, 2023); *Code of Ethics for Emergency Physicians*, American College of Emergency Physicians, https://www.acep.org/patient-care/policy-statements/code-of-ethics-for-emergency-physicians/ (accessed Apr. 14, 2023); *Ethics Manual*, 7th ed., American College of Physicians, https://www.acponline.org/clinical-information/ethics-and-professionalism/acp-ethics-manual-seventh-edition-a-comprehensive-medical-ethics-resource/acp-ethics-manual-seventh-edition (accessed Apr. 14, 2023).

107 See, eg Counsel on Ethical and Judicial Affairs, *Rules for Review of Membership*, American Medical Association, https://www.ama-assn.org/councils/council-ethical-judicial-affairs/ceja-rules-review-membership (accessed Apr. 14, 2023); *Procedures for Addressing Charges of Ethical Violations and Other Misconduct*, American College of Emergency Physicians, https://www.acep.org/life-as-a-physician/ethics--legal/ethics/procedures-for-addressing-charges-of-ethical-violations-and-other-misconduct/ (accessed Apr. 14, 2023). See also Steven F. Seidman, *Professional Misconduct and Ethics*, 34 Clin. Perinatol. 461 (2007).

108 Interview with L (July 11, 2022) (on file with author).

